# Different Effects of Soy and Whey on Linear Bone Growth and Growth Pattern in Young Male Sprague-Dawley Rats

**DOI:** 10.3389/fnut.2021.739607

**Published:** 2021-11-24

**Authors:** Meytal Bar-Maisels, Chen Menahem, Yankel Gabet, Sahar Hiram-Bab, Moshe Phillip, Galia Gat-Yablonski

**Affiliations:** ^1^The Jesse Z and Sara Lea Shafer Institute for Endocrinology and Diabetes, National Center for Childhood Diabetes, Schneider Children's Medical Center of Israel, Petach Tikva, Israel; ^2^Laboratory for Molecular Endocrinology and Diabetes, Felsenstein Medical Research Center, Petach Tikva, Israel; ^3^Sackler Faculty of Medicine, Tel Aviv University, Tel Aviv, Israel

**Keywords:** soy, growth plate, whey, catch up, micro CT, IGF-I, BMD

## Abstract

The aim of this investigation was to determine the better protein for supporting optimal linear growth, as the exact composition and benefits of specific dietary proteins in supporting linear growth is unknown. In the current study, we compared the effect of soy and whey proteins, both proteins contain all essential amino acids and are considered the best proteins in their categories. Young male rats were subjected to multiple feeding protocols using iso-energetic diets containing soy or whey as the sole protein source. The rats were allowed to eat *ad libitum* for 11, 24, or 74 days in the first set of experiments, and the soy group was pair-fed to the whey group in the second set. The differences in weight gain, food consumption, and humeri length of the soy group that were greater at the beginning of the *ad libitum* experiments lessened over time. Pair-fed experiments revealed that the increased weight and humeri length resulted from the differences in food consumption. However, other parameters were protein specific. Bone quality, which was better in the soy group at 24 days, was matched by the whey group and even surpassed that of the soy group in the long-term experiment, with a significantly greater bone mineral density, cortical thickness, and growth plate. Although in the short term the levels of insulin like growth factor (IGF)-I were similar between the groups, IGF-I increased with age in the whey group, and the levels at the long-term experiment were significantly higher compared to the soy group. Furthermore, using the pair fed setup made it clear that when the difference in food consumption were no longer playing part, whey was more efficient in increasing IGF-I. There were no indications of metabolic sequelae. Although the use of soy is gaining in popularity as a sustainable protein, our findings indicate a better effect of whey on linear growth by leading to slower growth with better-organized epiphyseal growth plates and bone quality.

## Introduction

Linear growth is a result of a complex system of interactions between genetics, epigenetics, and environmental factors (especially stress and nutrition). The association between nutrition and linear growth in children is well-recognized and documented in studies performed in underprivileged countries as well as in studies on the secular trend in height in European countries (1, 2). A study designed to explore the main correlates of male height in 105 countries (Europe & overseas, Asia, North Africa, and Oceania) with an average consumption of 28 protein sources and seven socioeconomic indicators concluded that nutrition and genetics are the strongest correlates of adult height (3). Intake of protein from milk products (dairy proteins), followed by total protein and animal protein (meat and eggs) consumption emerged as the most significant nutritional correlates of stature in most countries examined (3). The results of that study indicated that plant-based diets are not able to provide the optimal stimuli for physical growth, even if the intake of total protein and total energy are adequate. In fact, a difference of 10 cm in average male height (174 vs. 184 cm) was identified between nations relying upon the surplus of plant and animal proteins, respectively, pointing to the importance of protein quality (3).

Plant-based diets are sometimes considered a dietary strategy for maintaining good health, for protecting against inflammatory conditions, and for managing pathological conditions ranging from metabolic syndrome (including obesity, diabetes, and cardiovascular risk) to cancer. The use of plant-based protein isolates in food formulations has become a focus of interest due to greater sustainability, lower production costs, and a lower ecological footprint compared to animal-based ones. Although the average male height was found to correlate most negatively with proteins from rice and legumes (including soy) (3), the evidence of the differences in the effect of plant-based diets compared to animal-based diets on linear growth in children is mostly correlative, and the effect of plant-based diets on linear growth has not been tested in depth.

Proper growth and development in children are considered as markers for good health. Linear growth, driven by chondrocytes, is subject to regulation by numerous local and systemic factors, many of which are responsive to nutritional cues. The process of linear growth involves the sequential replacement of chondrocytes located in the cartilaginous growth center of the long bones [the epiphyseal growth plate (EGP)] by osteoblasts, a process regulated by complex interactions among hormones, local growth factors, and components of the extracellular matrix (ECM). Endochondral ossification begins with the proliferation of early chondrocytes (in the reserve zone), followed by their alignment in columns (in the proliferation zone), and, finally, their maturation into hypertrophic chondrocytes (in the hypertrophic zone). The hypertrophic cells then cease dividing, increase in volume by 5- to 10-fold, and boost the deposition of ECM components, mostly collagens and proteoglycans, and the secretion of matrix vesicles that serve as centers of mineralization. Thereafter, the chondrocytes undergo either programmed cell death, with calcification of the ECM, enabling the invasion of blood vessels and osteoblasts or transdifferentiating to endochondral osteoblasts. Bone tissue replaces the cartilage scaffold as a result of this chain of events. Proper elongation of the skeleton requires that endochondral ossification and bone modeling be tightly synchronized.

Although it is well-known that fasting and food restriction impair the rate of longitudinal bone growth and reduce the height of the EGP (2), the best food for supporting linear growth remains uncertain. Apart from the role in providing building blocks for cellular growth, proteins may also act as regulatory agents by affecting insulin like growth factor (IGF)-I and calcium absorbance and by altering the gut microbiome. Indeed, our previous studies have shown that the source of the consumed protein may affect proper growth (4). Our comparisons between two high-quality proteins of milk origin (whey and casein) revealed significant differences in linear growth and microbiome composition (4, 5).

In the current study, we compared the effect of soy and whey proteins on linear growth and bone strength in young fast-growing male rats. Both proteins contain all essential amino acids (EAA) and are considered the best proteins in their categories according to the protein digestibility-corrected amino acids score (PDCAAS). Whey is especially rich in branched amino acids (BCAA) as well as sulfur-containing amino acids (6) and is graded as the best protein source according to its essential amino acid score and PDCAAS (7). Soy ranks second after whey as a complete food protein and is the most popular plant proteins utilized in the production of newborn formulas and dietary supplements. Given that soy-based formulas are the formulas of choice for children with food allergies and for children whose parents opt to avoid food from animal-derived products for various reasons, it becomes all that more important to compare the effect of soy and whey proteins on linear growth.

## Methods

### Animals

All experiments were performed on pre-pubertal 26-d-old male Sprague-Dawley rats (Envigo Laboratories Ltd., Jerusalem, Israel). The approval of the Tel Aviv University Institutional Animal Care and Use Committee to which the Felsenstein Medical Research Center (FMRC) is affiliated was obtained before the experiments were initiated (committee protocol approval number 01-20-062). All of the animals were kept under the same experimental conditions: mean ambient temperature 23 (±1) °C, mean relative humidity 50 (±2) %, 12 h light/dark cycle, and lights off at 19:00 h. They all had free access to unfiltered regular tap water and were fed one of the custom-made commercial diets (Supplementary Table 1). The animals were kept two in a cage at the animal care facility of the FMRC or in single cages to allow monitoring of food intake during the catch-up and the pair-fed experiments. The animals were observed daily, and none showed signs of disease throughout the study apart from restlessness and slight aggressiveness during the food-restriction period.

### Feeding Regimens

The diets were iso-energetic and contained either soy protein (TD190912) or whey (TD190911; Teklad, Envigo Diets, Madison WI, USA) as the sole protein source (Supplementary Table 1). All other ingredients (cornstarch, carbohydrate, cellulose, fat, vitamins, and minerals) were identical. In the first *ad libitum* (AL) set of experiments, the rats were given free access to food and water for 11, 24, or 74 days. There were six animals per group in the short-term experiments, and eight animals per group in the 74-day experiments. In the second set of experiment (Pair-fed experiment) (*n* = 8), the amount that animals from whey group consumed was measured each day (during the 24 days of the study) and the same amount of food was then given to the pair-fed soy group the following day. To allow precise matching of food intake, the pair-fed group was started 1 day after the whey group. In the catch-up experiment, one group was fed AL with regular rat chow (TD 2918) (AL group, *n* = 6), and the restricted group was fed 60% of the normal daily intake of the same regular rat chow for 10 days (4). On day 10, the restricted group was further divided into one group that was fed the soy diet and the other group that was fed the whey diet for an additional 1 or 14 days (*n* = 6 in each group) with no restrictions. Experimental design is depicted in Supplementary Figure 1. All of the rats were euthanized by CO_2_ inhalation at the end of the experiments.

### Glucose Measurement and Serum Analysis

An intraperitoneal (i.p.) glucose tolerance test (GTT) was performed several days before the termination of the 74-day experiment. Animals were fasted for 6 h, and a glucose solution (1 g glucose/kg) was injected i.p. Blood glucose was measured by a portable glucometer (Contour plus, Ascensia Diabetes Care Holdings AG, Switzerland) in blood samples drawn by a needle puncture from a tail vein before and at 15, 30, 60, and 120 min post-glucose injection.

Fasting glucose levels were measured on a portable glucometer and assayed at the last day of the long-term experiment, in animals that were fasted for 12 h. The rats were euthanized by CO_2_ inhalation at the end of this experiment, and blood was collected by cardiac puncture. Serum was separated by centrifugation at 1,500 RPM (239^*^g) in a Rotina 46R centrifuge (Hettich Zentrifugen, Apeldoorn, the Netherlands) for 10 min at 4°C and stored at −70°C. Chemical analysis of the samples was performed by American Medical Laboratories, Israel (AML), and the results were compared to the control values supplied by AML. Serum levels of insulin-like growth factor-I (IGF-I), were determined using a commercial kit according to the manufacturer's recommendations (Quantikine Mouse/Rat IGF-I assay kit, detection limit 8.4 pg/ml [cat. no. MG100, R&D Systems, Minneapolis, MN, USA]).

### Histological Staining and Measurement of Growth Plate Height

The humeri of the euthanized animals were carefully removed, cleaned, and measured for length with a digital caliper. They were then fixed in 4% neutral buffered formalin for 48 h at room temperature, decalcified with Surgipath Decalcifier II (Leica Biosystems Richmond, Inc. USA) for several hours (depending upon the age of the animal), dehydrated through graded ethanol series (70, 95, and 100%), and stabilized by two sequential changes of chloroform for paraffin embedding. Histological studies and EGP height measurements were performed on deparaffinized sections of 6 μm thickness that had been stained with hematoxylin-eosin and Alcian blue. The height of the EGP was measured by drawing a straight line from the apical border of the reserve zone cells to the lower border of the mineralized cartilage. The findings presented here represent the average of at least five measurements per each section. The slides were photographed under an Olympus BX40 microscope equipped with an Olympus DP71 camera (Olympus Optical Co. GmbH, Hamburg, Germany), and analyzed with Image-Pro software (version 4.5.1.22, Media Cybernetics, Inc., Rockville, MD, USA).

### μCT Analysis

The humeri were kept in 4% neutral buffered formalin for 48 h at room temperature and then stored in 70% ethanol. The entire right humerus was scanned with a micro-computerized tomographic (μCT) system (μCT50, Scanco Medical AG, Switzerland). The scans were acquired at 90 kVp, 200 μA, and 1,000 ms for energy, intensity, and integration time, respectively, generating images with an isotropic nominal resolution of 17.2 μm. Two-dimensional (2D) CT images were reconstructed in 2,048 × 2,048 pixel matrices by means of a standard convolution-backprojection procedure (Scanco uct_reconstruction v6.1). A 3D Gaussian filter was used to attenuate the background noise in the volumes (σ = 0.8; support = 1). The scans were segmented by a global thresholding procedure (trabecular attenuation = 130; cortical attenuation = 200 in permille of the total gray value range). Morphometric parameters were determined with a direct 3D approach (8) in three different pre-selected analysis regions by means of customized software developed on the proprietary Image Processing Language v5.15 (Scanco Medical). We measured humerus length and bone volume fraction (BV/TV, %) (9) along the entire bone. In the cortical bone, we used a 1-mm-height diaphyseal segment starting at the 6th tenth of the total length (slightly distal to the midshaft). Cortical measurements included total area (Tt.Ar, mm^2^), cortical area (Ct.Ar, mm^2^), cortical area fraction (Ct.Ar/Tt.Ar, %), and cortical thickness (Ct.Th, mm). To analyze the trabecular bone, we used the secondary spongiosa of the proximal metaphysis of the humerus that had been manually separated from the cortical bone by tracing the endosteal surface on the axial 2D tomographic slices. The measurements included trabecular bone volume fraction (BV/TV, %), trabecular number (Tb.N, mm^−1^), trabecular thickness (Tb.Th, mm), and trabecular separation (Tb.Sp, mm).

### Statistical Analysis

Data are presented as mean ± standard deviation (SD). We used the One-Sample Kolmogorov-Smirnov Test to test the null hypothesis that distribution of the parameters is normal; all *P*-value were non-significant (*p* > 0.05), therefore all parameters have normal distribution and the significance of differences between experimental groups was determined using Student's *T*-test. Levene's test for equality of variance was used to check equal variance and we used the *P*-value accordingly. Differences were considered statistically significant at *p* < 0.05.

## Results

### Effect of Soy vs. Whey on Linear Growth (Short-Term AL Feeding)

**Figure 1:**
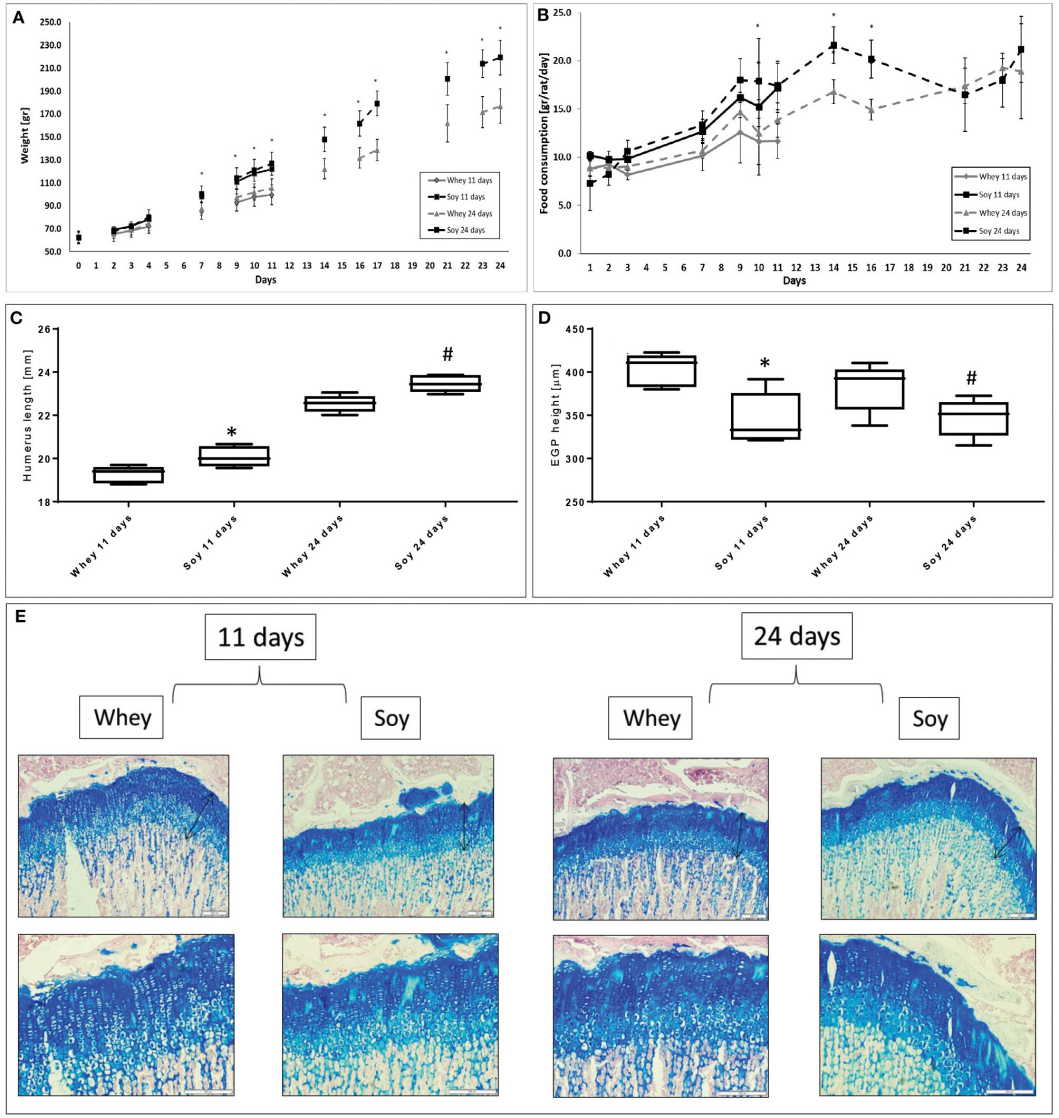
Differential effect of soy and whey diets on growth during 11 or 24 days of ad libitum feeding. **(A)** Body weight, **(B)** food consumption, **(C)** humerus length, **(D)** EGP height, and **(E)** representative stained sections of the EGP (upper panel-magnification ×10; lower panel -magnification X 20). Statistical analysis was done by Student's *T*-test. The asterisk (*) within the graphs designates significant differences at *p* < 0.05 for whey vs. soy at 11 days; The pound sign (#) within the graphs designates significant differences at *p* < 0.05 for whey vs. soy at 24 days. The black arrows indicate the height of the growth plates. **(C,D)** The box plot shows the minimum, first quartile, median, third quartile, and maximum in humerus length **(C)** and EGP height **(D)** after 11 and 24 days of free feeding. EGP, epiphyseal growth plate.

Young male Sprague-Dawley rats were fed AL for either 11 or 24 days in order to investigate whether there is any difference between the effects of the soy and whey diets on growth parameters (Figure 1A). The weight gain of the soy-fed rats was greater compared to the whey-fed rats from the beginning to the end of the study in both experiments. Food consumption of the soy group was greater until day 16 of the experiment after which the difference between the two groups diminished considerably (Figure 1B). The humeri of the soy-fed rats were significantly longer in both experiments (Figure 1C), however, the EGP height was greater in sections taken from the whey-fed animals (*p* < 0.05; Figures 1D,E).

### Effect of Soy vs. Whey on Linear Growth (Pair-Fed Feeding)

**Figure 2:**
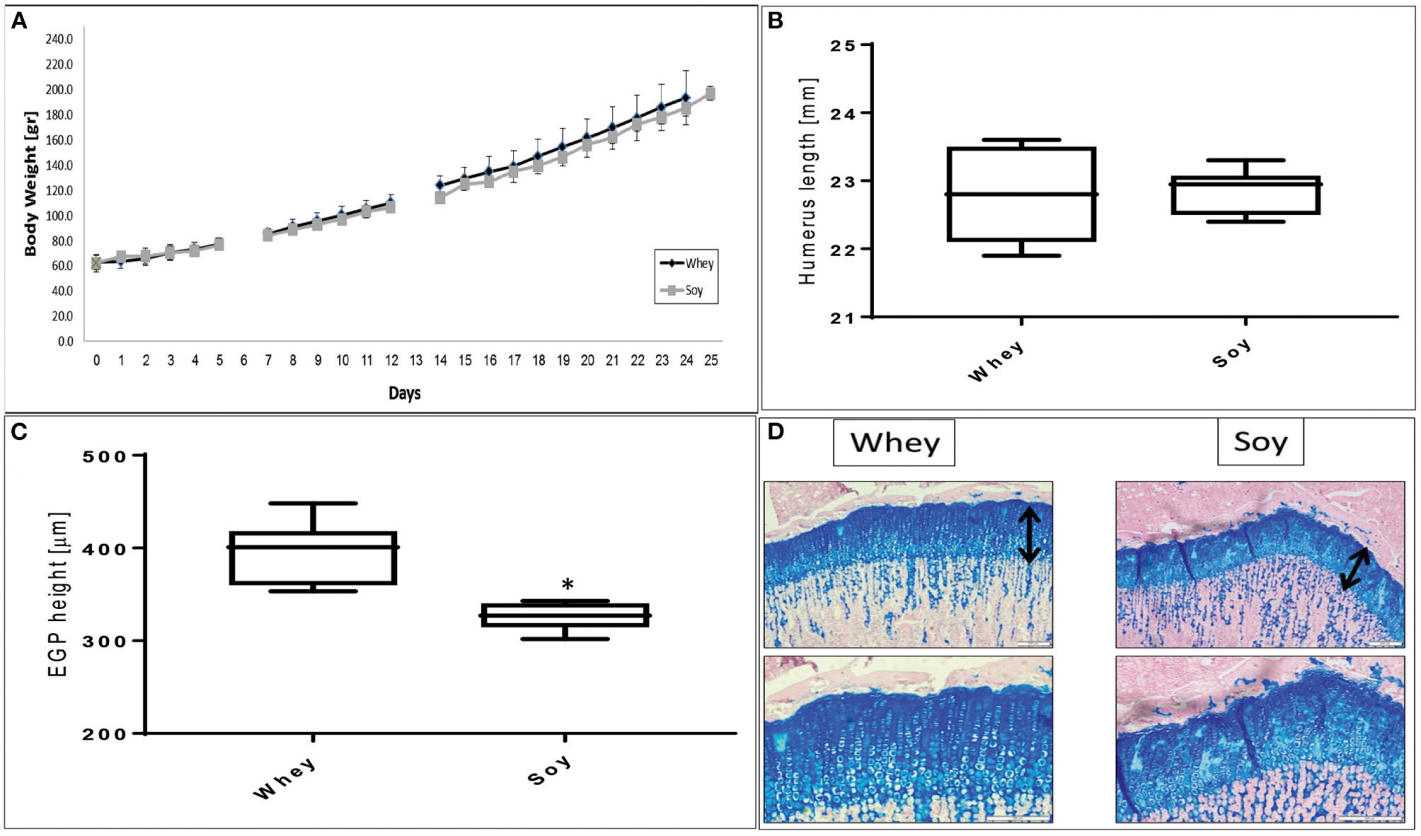
Pair-fed experiment. **(A)** Animal body weight, **(B)** humerus length, **(C)** EGP height, and **(D)** representative stained sections of the EGP (upper panel-magnification ×10; lower panel magnification X 20). Values are means ± SD. Statistical analysis was done by Student's *T*-test. The asterisk (*) within the graphs designates significant differences at *p* < 0.05. The black arrows indicate the height of the growth plates. **(B,C)** The box plot shows the minimum, first quartile, median, third quartile, and maximum of humeru length **(B)** and EGP height **(C)** after 24 days of pair-fed feeding. EGP, epiphyseal growth plate.

We performed a pair-fed study in which the amount of food provided to the soy-fed group was matched to that of the whey-fed group on the day before in order to determine if the different effect on growth was due solely to the difference in food consumption. It emerged that the weight gain in both groups was similar (Figure 2A). While the humerus length at the end of the experiment was not significantly different between groups (Figure 2B), the height of the EGP was significantly greater in the whey group compared to the soy group (*p* < 0.05; Figures 2C,D).

### Effect of Soy vs. Whey on Linear Growth (After Food Restriction)

**Figure 3:**
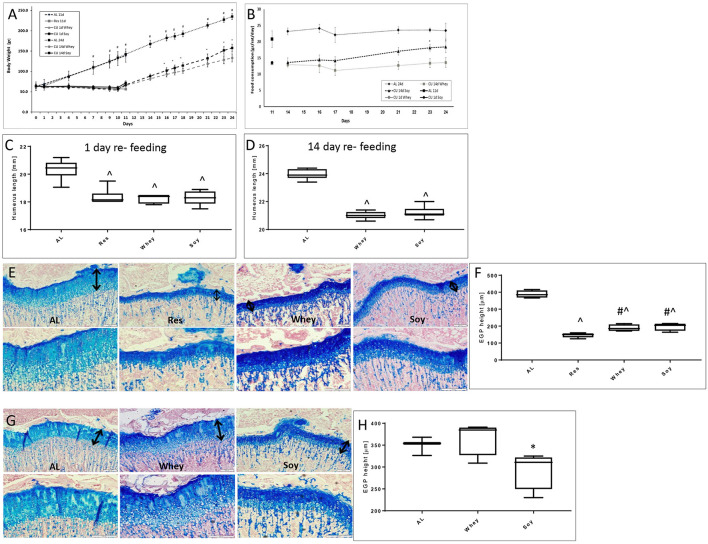
Catch-up growth experiment. **(A)** Animal body weight, **(B)** food consumption, **(C)** humerus length 1 day refeeding, **(D)** humerus length 14 days refeeding, and **(E)** representative stained sections of the EGP at 1 day refeeding (upper panel magnification ×10; lower panel magnification X 20); **(F)** EGP height at 1 day of refeeding. **(G)** Representative stained sections of the epiphyseal growth plate (EGP) at 14 days refeeding (upper panel magnification ×10; lower panel magnification X 20). The black arrows indicate the height of the growth plates. **(H)** EGP height at 14 days refeeding. Statistical analysis was done by Student's *T*-test. The asterisk (*) within the graphs designates significant differences at *p* < 0.05 for soy vs. whey. The pound sign (#) within the graphs designates significant differences at *p* < 0.05 for Res vs. Whey/Soy; The caret sign (∧) within the graphs designates significant differences at *p* < 0.05 for AL vs. Res/Whey/Soy. **(C,D,F,H)** The box plots show the minimum, first quartile, median, third quartile, and maximum of humerus length after 1 **(C)** or 14 days refeeding **(D)** and EGP height at 1 **(F)** or 14 days of refeeding **(H)**. EGP, epiphyseal growth plate; AL, *ad libitum*; RES, food restriction; CU, re-fed group, showing catch up growth.

After a period of growth attenuation, the removal of the growth inhibitory factor is usually followed by spontaneous catch-up growth (10, 11). A permanent growth deficit occurs when recovery is incomplete, leading to short stature. In view of the different effects of the soy and whey diets on linear growth when fed AL, we further examined whether the type of protein ingested during the re-feeding period will affect the efficiency of the catch-up growth process. Specifically, the animals were food restricted for 10 days and then re-fed for 1 or 14 days with either the soy or the whey diets. Soy led to more rapid weight gain, while whey led to a greater EGP (Figures 3A–H).

### Effect of Soy vs. Whey on Linear Growth (AL Feeding, Long-Term Follow Up)

**Figure 4:**
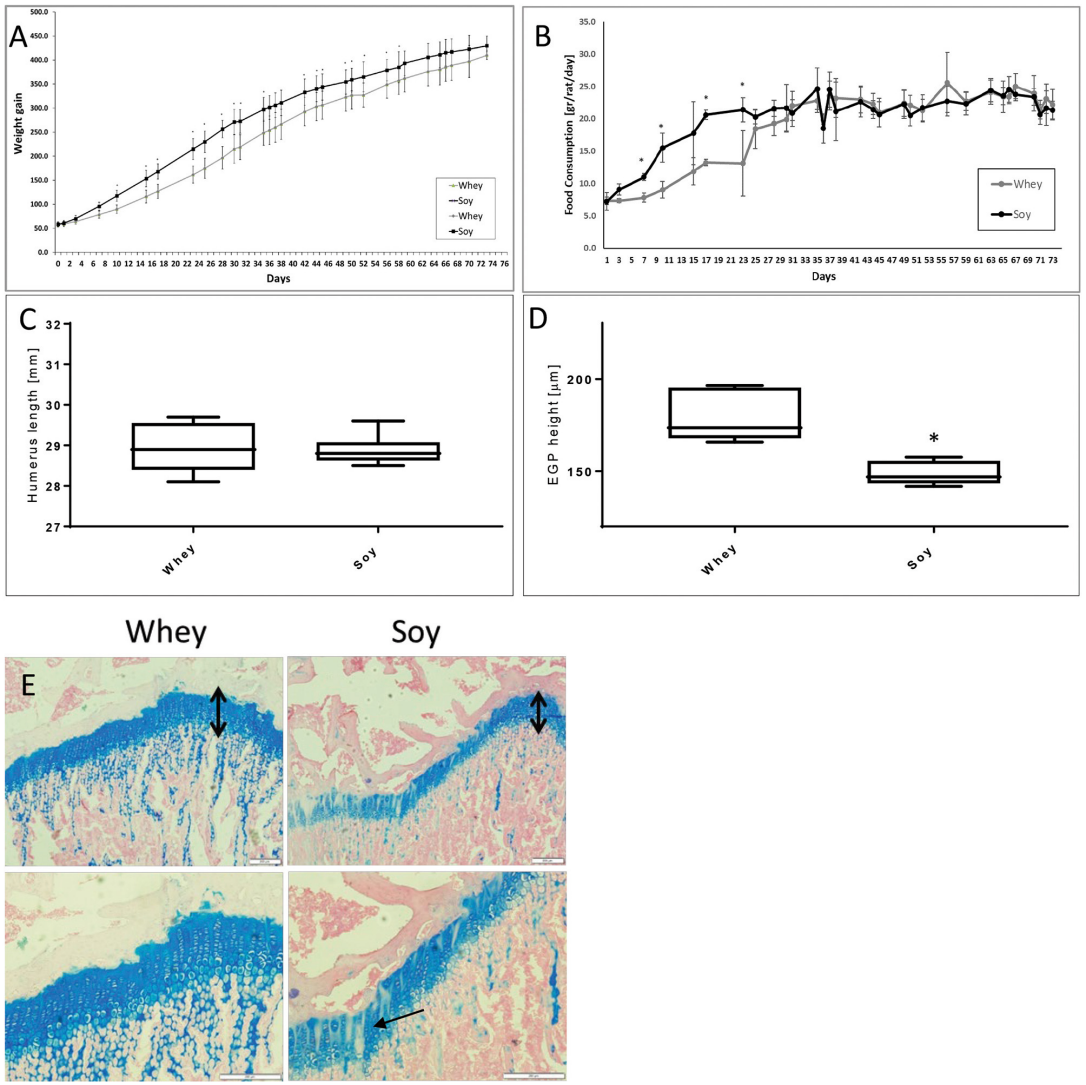
Long-term experiment. **(A)** Animal body weight, **(B)** food consumption, **(C)** humerus length, **(D)** EGP height, **(E)** Representative stained sections of the epiphyseal growth plate (EGP) (upper panel magnification ×10; lower panel magnification X 20) (upper row magnification ×10, lower row magnification ×20). The black arrows indicate the height of the growth plates. Note the better organization of the EGP in the whey group (marked with arrows). Statistical analysis was done by Student's *T*-test. The asterisk (*) within the graphs designates significant differences at *p* < 0.05 for Soy vs. Whey. **(C,D)** The box plot shows the minimum, first quartile, median, third quartile, and maximum in humerus length **(C)** and EGP height **(D)** after 74 days free feeding. EGP, epiphyseal growth plate.

We performed an additional AL study to check whether the different growth patterns that we had observed in both the AL and catch-up models will translate into differences in bone length when reaching adult size. The rats were randomized to eat one of the two diets AL after which they were followed up to the age of 100 days, an age at which, according to previous publications, bone length reaches its final length and the subsequent changes in length are minimal (12). The results (Figures 4A,B) showed that the differences in weight and food consumption that were apparent at the beginning of the study (and which matched those in the short-term experiments), were no longer apparent after 59 and 23 days, respectively. The weight, food consumption, and the length of the humeri (Figure 4C) were indistinguishable at the age of 100 days. However, EGP height was still significantly greater in the whey-fed group (Figures 4D,E). Moreover, EGP seemed to be better organized (Figure 4E), and the cell density in columns was greater in the whey group.

### Serum Analysis

We checked to see if there had been any effect of diet consumed on metabolic parameters in the long-term after having observed that the growth pattern, especially with regard to weight gain, was more robust in the soy-fed animals at the beginning of the study. GTT performed several days before sacrifice showed no significant differences between the groups (Supplementary Figure 2). The fasting glucose assessment, at the last day of the long-term experiment showed no significant differences between the groups (soy group 81 ± 4.5 mg/dL, whey group 82.5 ± 2.9 mg/dL; *p* = 0.44). Analysis of the blood samples taken at sacrifice showed that all values were within the normal range for Sprague-Dawley rats (Table 1; normal range provided by AML Israel Ltd.), and there was no evidence of interference in either kidney or liver activity. Interestingly, the soy-fed animals showed a lower level of total cholesterol (by 15%), with no comparable effect on triglyceride levels.

**Table 1:** Serum analysis in male rats after 74 days with soy or whey diets (values are presented as average ± SD).

	**Units**	**Whey**	**Soy**	***p*** **-value**	**Normal range**
Creatinine	mg/dl	0.45 ± 0.04	0.52 ± 0.06	0.050	0.27–0.65
Urea	mg/dl	36.31 ± 2.30	36.54 ± 2.43	0.86	29.93–59.17
SGOT	IU/L	126.57 ± 38.26	171.71 ± 2.43	0.065	57–210
SGPT	IU/L	37.14 ± 4.87	45.42 ± 9.90	0.08	30–106
Cholesterol	mg/dl	108.57 ± 17.11	92.71 ± 8.06	0.055	79.18–137.3
Trig	mg/dl	58.43 ± 15.5	59.57 ± 10.08	0.87	21–86
Total P	g/dl	6.42 ± 0.18	6.93 ± 0.26	0.001	5.92–7.46
Albumin	g/dl	4.4 ± 0.16	4.7 ± 0.20	0.01	3.96–4.73
Glob	g/dl	2.02 ± 0.10	2.23 ± 0.27	0.10	1.69–3.01
T. Bil	mg/dl	0.04 ± 0.01	0.03 ± 0.01	0.35	0.03–0.18
Alk Phos	IU/L	111.28 ± 16.59	107.29 ± 7.08	0.57	81.42–197.75
Calc	mg/dl	11.9 ± 0.053	12.01 ± 0.36	0.65	9.92–12.28
Phos	mg/dl	13.58 ± 1.08	14.1 ± 1.40	0.46	8.13–12.11
Na	mmol/L	146.4 ± 1.51	147 ± 1.73	0.52	142–147
K	mmol/L	9.01 ± 0.62	9.37 ± 1.13	0.48	5.3–7.3
Cl	mmol/L	98.71 ± 1.38	99.28 ± 1.25	0.43	94–101

*Statistical analyses was done by Student's T-test. Normal range values per Sprague-Dawley rat were provided by AML Ltd. SGOT, serum glutamic oxaloacetic transaminase; SGPT, serum glutamic-pyruvic transaminase; Trig, triglycerides; Total P, total protein; Glob, serum globulins; T. Bil, total Bilirubin; Alk Phos, alkaline phosphatase; Calc, calcium; Phos, phosphate*.

### IGF-I Levels

IGF-I levels were not significantly different between the groups at the end of the short-term experiment, however when the differences in food consumption were excluded (pair-fed and long-term experiments) the serum level of IGF-I were significantly greater in the whey group (Table 2). IGF-I increased over time (i.e., with age) by 55% in the whey group (*p* = 0.001) and by 15% in the soy group (*p* = 0.03).

**Table 2:** Serum IGF-I levels (ng/ml).

	**Whey**	**Soy**	***p*** **-value**
24 days AL	1102.3 ± 161.7	1230.3 ± 85.1	0.2
24 days pair-fed	1508.5 ± 93.8	1229.1 ± 92.6	0.0003
74 days AL	1712.1 ± 239.9	1412.9 ± 146.4	0.03

*AL, ad libitum*.

### μCT Analysis

**Figure 5:**
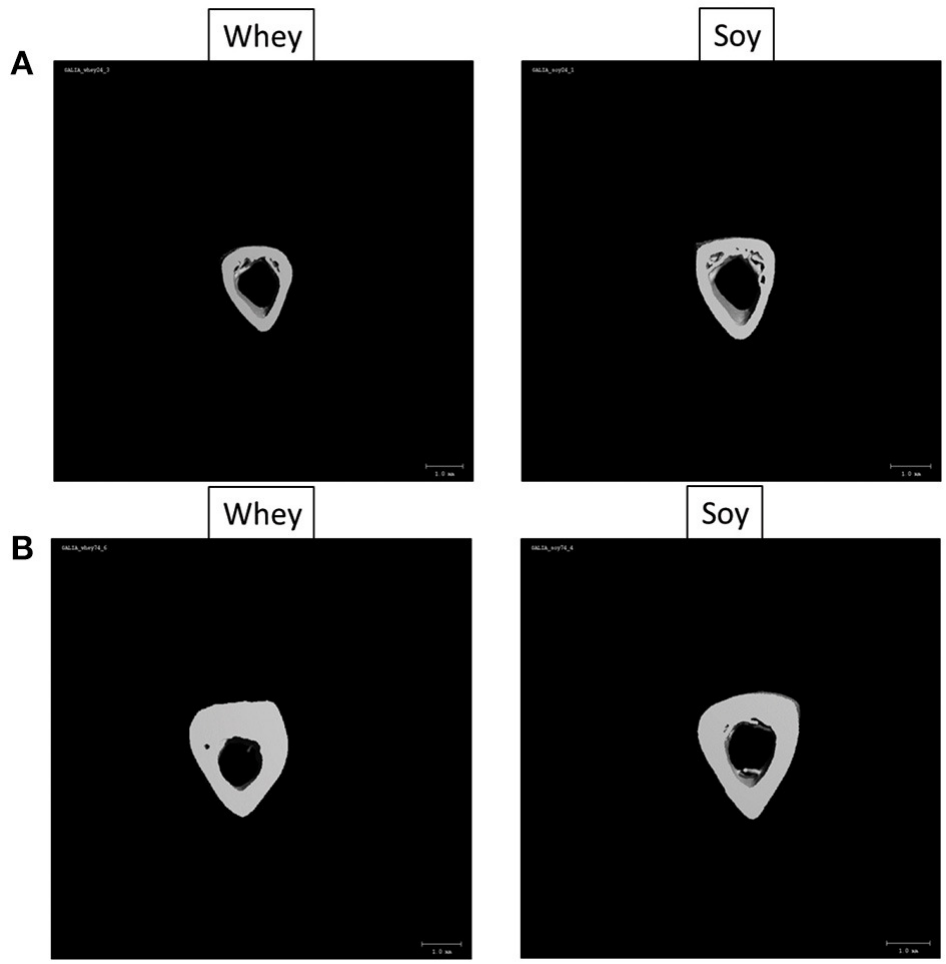
Three-dimensional cortical bone images obtained by μCT at **(A)** 24 days or **(B)** 74 days.

The long bones consist mainly of two different types of structures: the cortical bone, which forms the hard outer layer of the long bone, and the trabecular bone (spongy bone), which is less dense and less stiff, and has a higher surface area enabling high vascularization. μCT analysis, the gold standard for determining bone microstructure in animal models, was used to study the effect of the diets on humeri from both the 24-day and the 74-day experiments (Table 3). The humerus length of the soy group was greater in the short-term experiment, and the μCT analysis showed that the diaphyseal diameter (Dia.Dia) of the cortical bone was also greater, suggesting an accelerated radial growth. This likelihood is also supported by the greater moment of inertia (MOI) parameters, which predict the resistance of the bone to shear forces, in the soy-fed rats (Table 3 and Figures 5A,B). At age 100 days, after 74 days of feeding, we found that all the parameters that showed improved values at 24 days in the soy group were no longer different between the diet groups. Indeed, the whey group reached the same values for bone length, Dia.Dia and all MOI values, as those of the soy group at 74 days. Moreover, the whey-fed animals had superior micro architectural parameters in the full humerus (%BV/TV, vBMD), mainly due to improved cortical parameters. The Ct.Ar/Tt.Ar was greater in the whey group because of the thicker cortex (Ct.Th) at the expense of the medullary cavity diameter (Med.Dia) (Table 3 and Figures 5A,B).

**Table 3:** Bone parameters (μCT) in male Sprague-Dawley rats after 24 or 74 days.

	**Whey 24 d**	**Soy 24 d**	***p*** **-value**	**Whey 74 d**	**Soy 74 d**	***p*** **-value**
	**(***n*** = 6)**	**(***n*** = 6)**		**(***n*** = 7)**	**(***n*** = 8)**	
			**Soy vs. Whey**			**Soy vs. Whey**
**(A) Full humerus length (mm)**	22.55 ± 0.38	23.55 ± 0.37	0.001	28.37 ± 0.75	28.59 ± 0.37	0.49
% BV/TV	70 ± 6	65 ± 5	0.14	71 ± 1	66 ± 1	0.002
Volumetric bone mineral density (vBMD) [mg HA/ccm]	354.57 ± 74.48	367.73 ± 56.58	0.74	651.98 ± 19.22	586.08 ± 37.53	0.011
**(B) Cortical bone parameters**						
Tt.Ar (mm^2^)	3.88 ± 0.23	4.46 ± 0.38	0.012	6.04 ± 0.50	5.85 ± 0.58	0.49
Ct.Ar (mm^2^)	2.46 ± 0.63	2.99 ± 0.47	0.13	4.7 ± 0.33	4.19 ± 0.36	0.017
Ct.Ar/Tt.Ar	0.63 ± 0.14	0.67 ± 0.09	0.56	0.78 ± 0.03	0.72 ± 0.02	0.001
Ct.Th (mm)	0.4 ± 0.16	0.47 ± 0.12	0.39	0.65 ± 0.02	0.59 ± 0.04	0.007
Dia.Dia (mm)	2.22 ± 0.07	2.38 ± 0.10	0.01	2.77 ± 0.11	2.73 ± 0.14	0.49
Med.Dia (mm)	1.33 ± 0.23	1.36 ± 0.18	0.80	1.3 ± 0.12	1.45 ± 0.11	0.03
**(B1) MOI parameters**						
I min (mm^4^)	0.9 ± 0.21	1.26 ± 0.28	0.03	2.49 ± 0.43	2.2 ± 0.34	0.15
Polar (mm^4^)	2.06 ± 0.45	2.88 ± 0.58	0.02	5.77 ± 0.86	5.26 ± 1	0.3
Areal (mm^3^)	0.87 ± 0.18	1.12 ± 0.15	0.03	1.82 ± 0.20	1.73 ± 0.21	0.41
**(C) Trabecular bone parameters**						
Tb.BV/TV	0.23 ± 0.13	0.25 ± 0.07	0.72	0.25 ± 0.06	0.22 ± 0.03	0.27
Tb.Th (mm)	0.1 ± 0.03	0.11 ± 0.01	0.84	0.1 ± 0.01	0.11 ± 0.004	0.35
Tb.N (mm^−1^)	1.79 ± 0.49	1.93 ± 0.25	0.56	1.88 ± 0.83	1.62 ± 0.37	0.46
Tb.Sp (mm)	0.65 ± 0.12	0.60 ± 0.06	0.37	0.68 ± 0.27	0.72 ± 0.15	0.75

*All values are mean ± SD. Bone length and cortical thickness and BV/TV of the distal bone were significantly different between the two diets. BV/TV, Bone volume/total volume; vBMD, bone volumetric bone mineral density; Tt.Ar, total area; Ct.Ar, cortical area; Ct.Ar/Tt.Ar, cortical area fraction; Ct.Th, cortical thickness; Dia.Dia, diaphyseal diameter; Med. Dia, medullary diameter; MOI, moment of inertia; I min, Minimum moment of inertia; Tb. Th, trabecular thickness; Tb.N, trabecular number; Tb.Sp, trabecular separation*.

The differences in growth pattern were better exemplified by analysis of age-induced changes in the μCT parameters (Table 4). Changes over time clearly showed that while many bone parameters were better in the soy group at the end of the short-term experiment, the whey group corrected most parameters of bone structure over time, leading to the same length and partially better cortical bone parameters.

**Table 4:** Age-dependent change in bone parameters (μCT) in male Sprague-Dawley rats depending upon the diet (values are ratio of 74/24 parameters and presented as percent change).

**Change**	**Whey**	**Soy**	***p*** **-value**
Total length	+26	+21	0.01
Tt. % BV/TV	+0.008	+0.02	0.65
vBMD	+83	+59	0.001
Dia.Dia	+24	+14	0.002
Med.Dia	+98	+6	0.07
Ct.Th	+62	+24	<0.001
MOI I min	+177	+173	<0.001
MOI polar	+180	+182	<0.001
MOI areal	+109	+55	<0.001
Tb.BV/TV	+8	−14	0.086
Tb.Conn.D	+19	−13	0.045
Tb.SMI	−20	+19	0.01
Tb.N	+4	−16	0.3
Tb.Th	NC	NC	0.85
Tb.Sp	+4	+20	0.4

*NC, no change; BV/TV, bone volume/total volume; vBMD, bone volumetric bone mineral density; Tt.Ar, total area; Ct.Ar, cortical area; Ct.Ar/Tt.Ar, cortical area fraction; Ct.Th, cortical thickness; Dia.Dia, diaphyseal diameter; Med.Dia, medullary diameter; MOI, moment of inertia; Conn.D, connectivity density; I min, minimum moment of inertia; SMI, structure model index; Tb.Th, trabecular thickness; Tb.N, trabecular number; Tb.Sp, trabecular separation*.

In the humeral proximal metaphysis, the trabecular bone showed no statistically significant differences between the groups at 24 and 74 days (Table 3). However, there was a distinct pattern of time-related changes in the trabecular parameters (Table 4). While the connectivity density (Conn.D) tended to decrease in the soy groups between 24 and 74 days of the diet, it tended to increase in the whey group (*p* = 0.045). There was a similar pattern for the trabecular BV/TV, although the difference in time-related changes was of borderline significant (*p* = 0.08).

These data showed that the skeletal response to the type of protein in the diet had a time/age dependency, with increased growth in the soy group during the first 3.5 weeks and increased growth in the whey group during the following 10.5 weeks.

## Discussion

The aim of this investigation was to determine the better protein for supporting optimal linear growth. The most interesting observation of our current study was the different effect of the two proteins on the growth pattern and humerus bone quality in an animal model. The differences in weight gain observed after 24 days of feeding were no longer apparent after 74 days of feeding. Bone quality, which seemed to be better in the soy group after 24 days of feeding was matched and even surpassed, by the whey group after an additional 50 days of feeding. In the long-term experiment, μCT analysis revealed a significant difference in bone mineralization between the groups, suggesting better biomechanical parameters in the whey group. We also observed a higher and better-organized EGP in the whey groups, with no significant differences in the trabecular compartment throughout the study. The effect on growth was similar in all of the study setups: the soy diet led to a more rapid weight gain and bone growth, while the whey diet led to slower growth with better outcomes. The effect of whey on growth was slower, maintaining a higher EGP for a longer time, similar to what we had found in a previous study in which we compared casein- and whey-based diets (4). Both serum analysis and GTT showed that in spite of the greater food consumption and weight gain at the beginning of the study, there were no indications for metabolic disease in either group: there was no effect on kidney or liver function, and all metabolic values were within the normal range for rats.

There is an increasing interest in plant-based foods due to both ecological and financial reasons. Production of plant-based foods requires less land and water and is associated with lower greenhouse gas emissions compared with animal-based foods. At the same time, however, plant-based proteins are considered as being of lesser quality with respect to their ability to increase both post-prandial muscle protein synthesis rates (13) and linear growth, as exemplified by the differences in male adult height (3). However, there is large variability in amino acid composition among different plant-based protein sources (14), and soy protein is among the few plant-based proteins that meet the requirements for EAA content and is therefore considered the best vegetarian protein (15). Given that the quality of soy proteins is considered as being superior to that of other plant proteins, we decided to compare it to whey in fast-growing young rats.

Soy consumption has historically been associated with Asian countries, however, the popularity of soy foods in the United States increased significantly after the approval by the Food and Drug Administration that soy protein has the ability to protect against cardiovascular diseases (16). Indeed, in the current study, cholesterol levels were significantly lower in the soy-fed rats (by 15%), although no effect on triglyceride levels was noted. This is in agreement with previous studies that showed that the intake of soy products resulted in a significant reduction in serum cholesterol concentration (by about 5%) in both humans (17) and rodents (18–20). A possible mechanism of the cholesterol-lowering effect of soy protein is its ability to modulate low-density lipoprotein receptor levels in the liver (20). The greater effect observed in our study compared to that cited in the literature may lie in the fact that we gave the animals purified soy protein, while a more complex diet had probably been given to the humans or to the animals in those studies.

A number of epidemiological and experimental studies claimed that soy has other health benefits, including its ability to mitigate obesity, diabetes, and related complications (21). In our study, no effect on glucose levels either during fasting or in response to glucose loading was found between the groups, which may be due to the young age of the animals. The only beneficial effect that we found in soy food was a reduction in cholesterol levels, described above, which indeed can be associated with reduced risk of cardiovascular diseases.

Several distinguishing features may account for the different effects of soy and whey on bone quality and linear growth in the long term:

1. The quality of a protein is primarily based upon EAA composition. EAA, defined as amino acids that cannot be synthetized by the organism and must be provided by food, are the building blocks for protein synthesis and, as such, they are required for growth. Both soy and whey contain all EAA. However, while the amino acid composition of whey is similar to that of muscle proteins and delivers the appropriate amino acid ratio upon digestion, the amino acid composition of soy has a shortage of specific amino acids, such as leucine, isoleucine, lysine, and methionine (14, 22). When matched for nitrogen content, soy reportedly stimulates protein synthesis to a lesser extent than whey (23–29). However, to the best of our knowledge, the effect of soy on linear growth and EGP has not been studied before.

The difference in protein quality between soy and whey is mostly due to soy's lower level of the BCAA leucine and the sulfuric amino acids methionine (15). BCAA are not only elementary components for building muscle and skeletal tissue, but they also stimulate protein synthesis in both animals and humans. BCAA regulate many key signaling pathways, the most classic of which is the activation of the mammalian target of the rapamycin complex 1 (mTORC1) signaling pathway. mTORC1 is an evolutionary-conserved multi-protein complex that coordinates a network of signaling cascades and functions as a key mediator of protein translation, gene transcription, and autophagy, and thus connects many diverse physiological and metabolic processes. Signal transduction through mTORC1, which is centrally involved in enhanced protein translation, is governed by an intracellular amino acid supply (30). Specifically, leucine, whose level in soy is only 58% of that in whey, was found to enhance mTORC1 signaling as well as repress proteasomal degradation (31–33), thus leading to activation of downstream signaling and subsequent stimulation of protein synthesis. As such, the leucine content of the ingested protein source forms a key characteristic that modulates activation of protein synthetic machinery after protein ingestion.

The non-proteinogenic functions of EAA should also be considered in order to better understand the physiological consequences of an insufficient intake of specific amino acids. This more notably concerns the sulfuric amino acids, methionine (Met) and cysteine (Cys) which are involved in methylation processes, participate in the control of oxidative stress, and affect metabolism and cell functions (34). The low content of Met in soy protein limits the latter's nutritive value. Met is the precursor of Cys, which is a constituent of glutathione and a precursor of taurine. The response to an insufficient Met supply has been reportedly associated with significantly reduced food intake and body weight gain, an increase in energy expenditure, and the down-regulation of genes involved in fatty acid and triglyceride synthesis in the liver, thus reducing its capacity to synthesize and export lipids to peripheral tissues (34).

Interestingly, the animals of the soy group in the current study showed a more rapid weight gain and increased food consumption in the short-term experiments. It may be that by using a relatively large amount of protein in the diets [28%; according to the AMDR (Acceptable Macro Nutrient Distribution Rate) protein content should be 10–35% from the total protein daily intake], the lower amounts of leucine and methionine were no longer an obstacle to growth. However, we still do not have an explanation for the increased food consumption, since the animals were provided with only one type of food.

2. Our results suggest that whey leads to better calcium absorption: both the soy and whey diets contained identical amounts of calcium, but the bone mineral density was higher in the whey group. Although some studies showed that phytic acid in soy-based diets could adversely affect mineral utilization (35), our soy diet did not contain phytic acid, thus we cannot contribute to the explanation for this effect.
3. IGF-I directly stimulates the proliferation and differentiation of EGP chondrocytes (36) as well as of osteoblasts (37) and increases trabecular and cortical bone formation. Both soy and whey diets have been shown to stimulate circulating IGF-I concentrations (4, 38), however, conflicting data made it very difficult to ascertain whether soy and whey similarly affect IGF-1 (39). In the current study, we compared diets that differed only in their protein source, thus focusing specifically on the protein- IGF-I connection. Although in the short term the levels of IGF-I were similar between the groups, IGF-I increased with age in the whey group, similarly to our previous findings (4), and the levels at the long-term experiment were significantly higher. Furthermore, using the pair fed setup made it clear that when the difference in food consumption were no longer playing part, whey was more efficient in increasing IGF-I.
4. Diet composition was shown to have an effect on the gut microbiome. The gut microbiota can influence the host by regulating nutrient and energy absorption, by producing vitamins and other useful metabolic byproducts, and by stimulating the host immune system at the gut lining. The microbiome has recently been identified as a factor that can influence bone quantity and bone quality (40, 41). In one study, the colonization of germ-free mice with normal gut microbiota led to normalization of bone mass, probably by affecting the immune system (42). Subsequent studies have shown the effect of the gut microbiome on bone, either through the effect on gut-derived serotonin (40), short chain fatty acids (SCFA) (43), or through the effect on osteoclasts (42). Diets composed of animal or plant constituents differentially alter the Firmicutes/Actinobacteria to Bacteroidetes ratio (21), with an animal-based diet preferentially promoting the abundance of Bacteroidetes and reducing Firmicutes compared to a plant-based diet. Soy dietary proteins were shown to alter the intestinal environment by affecting fermentation by gut microbiota and the generation of putrefactive compounds (44). However, there is currently no consensus on specific changes of gut microbiota by the soy protein, and a variety of results have been reported (21). Our previous analysis of the different effects of whey and casein on the gut microbiome showed that even proteins with high similarity could affect the gut microbiome in different ways (5).

*Limitations of the study*: Rats and humans are quite similar in physiology and anatomical structures, particularly the linear growth processes in both species that are composed of anatomically similar organs. Both rats and humans are omnivorous; therefore, they share strong similarities in dietary requirements. However, this study was performed on rats and not on children, and extrapolation of the findings to apply to children should be made with utmost caution. Another limitation of the study lies in the fact that only males were tested. This was due to the fact that males enter puberty later enabling a longer period of intervention (45), in the next studies, the effect on females should be completed.

## Conclusions

Using more plant-based proteins in the human diet and supporting sustainability of our planet is important. However, our results clearly point to a superior effect of whey on linear growth. Efforts are being made to develop soybean lines that overexpress methionine-rich proteins in order to improve the soy protein. Alternatively, the addition of methionine to a soymilk formula was shown to increase nitrogen retention of malnourished children (16). We have no explanation why the soy diet led to comparatively increased food consumption, weight gain, and linear growth in the short term, since our animals were given only one choice of diet. However, it may suggest that soy should be used in the first steps of re-feeding rehabilitation in malnourished children, leading to a more rapid weight gain, and that whey-based diets should be used in order to keep the growth potential and limit weight gain (4, 46). Alternatively, it may be possible that a combination of soy and whey would be a more beneficial approach. Creating protein blends seems to offer benefits over increasing the dose of proteins being consumed since protein blends can provide sufficient amounts of all essential amino acids and the benefits of the better of the two worlds.

## Data Availability Statement

The raw data supporting the conclusions of this article will be made available by the authors, without undue reservation.

## Ethics Statement

The animal study was reviewed and approved by Tel Aviv University Institutional Animal Care and Use Committee to which the Felsenstein Medical Research Center (FMRC) is affiliated. Approval was obtained before the experiments were initiated (committee protocol approval number 01-20-062).

## Author Contributions

GG-Y and MP: conceptualization. MB-M and CM: formal analysis. MP: financial resources. MB-M, SH-B, YG, and GG-Y: data curation. GG-Y: writing—original draft preparation, writing—review and editing, supervision, and project administration. All authors have read and agreed to the final version of the manuscript.

## Conflict of Interest

The authors declare that the research was conducted in the absence of any commercial or financial relationships that could be construed as a potential conflict of interest.

## Publisher's Note

All claims expressed in this article are solely those of the authors and do not necessarily represent those of their affiliated organizations, or those of the publisher, the editors and the reviewers. Any product that may be evaluated in this article, or claim that may be made by its manufacturer, is not guaranteed or endorsed by the publisher.
