# Supplementary material for: Different Effects of Soy and Whey on Linear Bone Growth and Growth Pattern in Young Male Sprague-Dawley Rats
Source: Front Nutr. 2021 Nov 24;8:739607. doi: 10.3389/fnut.2021.739607 (PMC8652289; doi:10.3389/fnut.2021.739607)
Supplement: Supplementary file 1 [file Data_Sheet_1.docx]

Supplementary Material


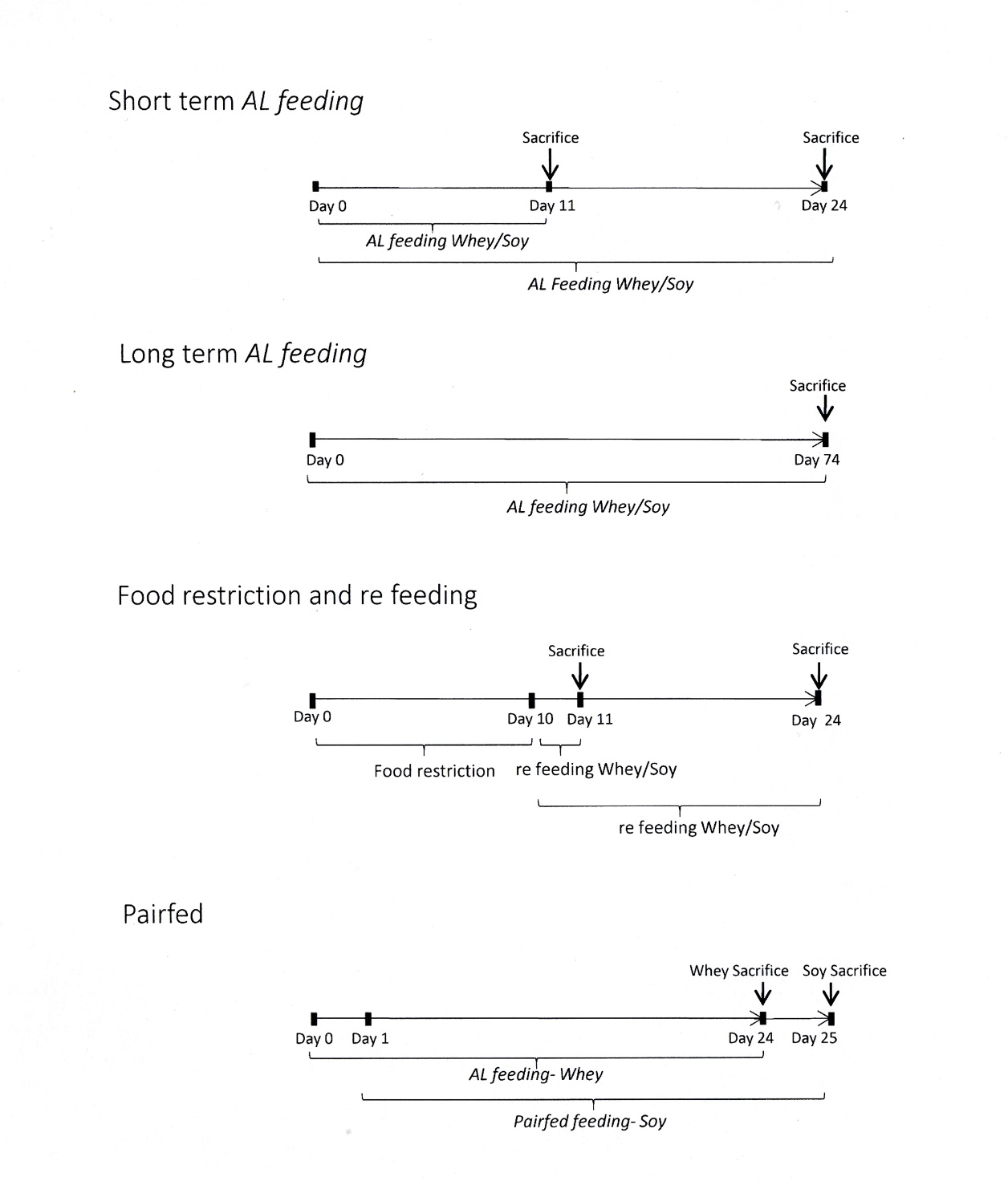


**Supplementary Figure 1:** Schematic presentation of the experimental design**.**


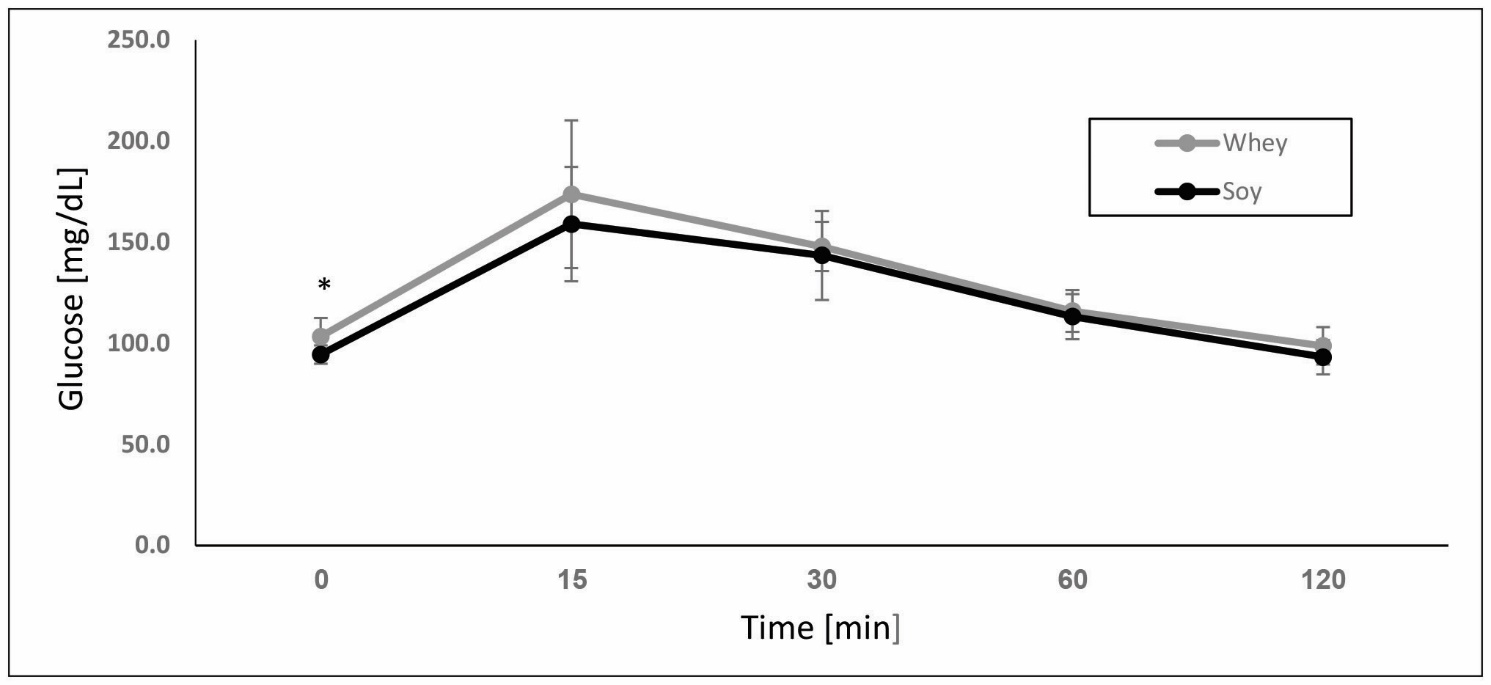


**Supplementary Figure 2.** The mean and standard deviation for the blood glucose values during the i.p. glucose tolerance test (GTT; 1 g glucose/kg) (n=8). The asterisk (*) within the graphs designates significant differences at *p*<.05 for Soy vs. Whey at the beginning of the study. There were no statistically significant differences between the groups in response to glucose load.

**Supplementary Table 1.** Macronutrient, vitamin, and mineral content of the experimental diets

|  | **TD190911 (Whey)** | **TD190912 (Soy)** |  |
| --- | --- | --- | --- |
| Energy (Kcal/g) | 3.3 | 3.3 |  |
| Carbohydrate (g/Kg) | 383 | 395 |  |
| Protein (g/Kg) |  |  |  |
| Whey | 232 |  |  |
| Soy |  | 231 |  |
| Fat (g/Kg) | 91 | 93 |  |
| Corn Starch (g/Kg) | 260 | 271.207 | |
| Cellulose (g/Kg) | 189.097 | 163.99 | |
| Minerals | | | |
| Calcium, g/kg | 9.5 | 9.5 | |
| Phosphorus, g/kg | 4.2 | 5.2 | |
| Sodium, g/kg | 3.5 | 4.6 | |
| Potassium , g/kg | 7.2 | 7.4 | |
| Chloride, g/kg | 3.1 | 3.1 | |
| Magnesium, g/kg | 1.0 | 1.2 | |
| Zinc, mg/kg | 69.1 | 82.6 | |
| Manganese, mg/kg | 20.5 | 25.0 | |
| Copper, mg/kg | 11.7 | 15.1 | |
| Iodine, mg/kg | 0.4 | 0.4 | |
| Iron, mg/kg | 70.9 | 105.3 | |
| Selenium, mg/kg | 0.29 | 0.29 | |
| Chromium, mg/kg | 1.94 | 1.94 | |
| Vitamins |  |  | |
| A (IU/g) | 15,000 | 15,000 | |
| B_1_ (thiamin), mg/kg | 17.8 | 17.8 | |
| B_2_ (riboflavin), mg/kg | 22.0 | 22.0 | |
| B_3_ (niacin), mg/kg | 99.0 | 99.0 | |
| B_6,_ mg/kg | 18.0 | 18.0 | |
| B_12,_ mg/kg | 0.03 | 0.03 | |
| C, mg/kg | 991 | 991 | |
| D, IU/g | 1,500 | 1,500 | |
| E, mg/kg | 100 | 100 | |
| Choline, mg/kg | 1,216 | 1,216 | |
| Biotin, mg/kg | 0.4 | 0.4 | |
| Folic acid, mg/kg | 2 | 2 | |
| Inositol, mg/kg | 110 | 110 | |
| K_3,_ mg/kg | 50 | 50 | |
| Pantothenic acid | 60 | 60 | |
